# Supplementary material for: Identification of photoperiod-induced specific miRNAs in the adrenal glands of Sunite sheep (Ovis aries)
Source: Front Vet Sci. 2022 Jul 22;9:888207. doi: 10.3389/fvets.2022.888207 (PMC9354845; doi:10.3389/fvets.2022.888207)
Supplement: Supplementary file 1 [file Data_Sheet_1.ZIP › Supplementary file/miRDeep2 file.zip!/miRDeep2 file/Novel_Structure/SP42A2/Novel_290.pdf]

Provisional ID	:	NC_019484.2_43018
Score total	:	2.1
Score for star read(s)	:	-1.3
Score for read counts	:	0
Score for mfe	:	1.8
Score for randfold	:	1.6
Score for cons. seed	:	
Total read count	:	54
Mature read count	:	54
Loop read count	:	0
Star read count	:	0

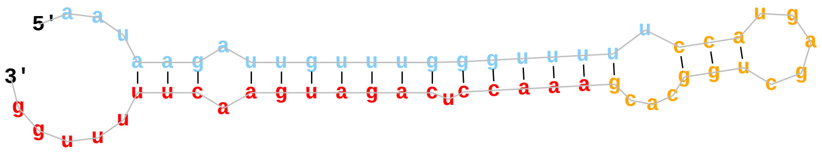

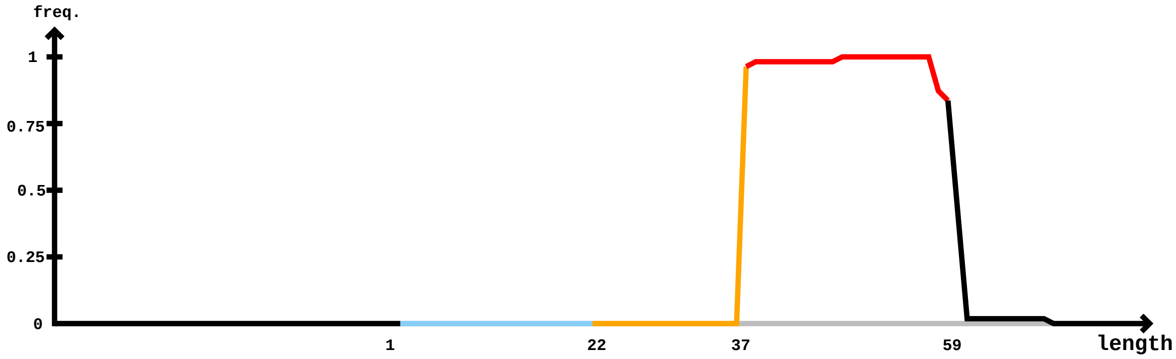


```
                                                      Star                             Mature
5'- ugcaugugugugauguguauuuauugggauggccaauaaagauuguuuggguuuuuccaugagcuggcacgaaaccucagaugaacuuuuuggcuaacccaauauuuaccagu   -3'    exp
    .((((.......))))(((..(((((((.(((((((.(((.(((((((((((((.(((.....)))...)))))).))))))).))).))))))).)))))))..)))....      reads      mm        sample
    .......................................................................aaaAcucagaugaacuuuuu......................      6          1         seq
    .......................................................................aaaccucagaugaacuuuuu......................      1          0         seq
    .......................................................................aaaccucagaugaacuuuuug.....................      1          0         seq
    .......................................................................aaaccucagaugaacuuuuugg....................      2          0         seq
    .......................................................................aaaAcucagaugaacuuuuugg....................      43         1         seq
    ........................................................................aaAcucagaugaacuuuuug.....................      1          1         seq
    .................................................................................ugaacuuuuuggcuaacccaau..........      1          0         seq
```
